# QNA-Based Prediction of Sites of Metabolism

**DOI:** 10.3390/molecules22122123

**Published:** 2017-12-01

**Authors:** Olga Tarasova, Anastassia Rudik, Alexander Dmitriev, Alexey Lagunin, Dmitry Filimonov, Vladimir Poroikov

**Affiliations:** 1 Institute of Biomedical Chemistry, 10 Building 8, Pogodinskaya Street, 119121 Moscow, Russia; rudik_anastassia@mail.ru (A.R.); a.v.dmitriev@mail.ru (A.D.); alexey.lagunin@ibmc.msk.ru (A.L.); dmitry.filimonov@ibmc.msk.ru (D.F.); vladimir.poroikov@ibmc.msk.ru (V.P.); 2 Pirogov Russian National Research Medical University, 1 Ostrovityanova Str., 117997 Moscow, Russia

**Keywords:** sites of metabolism, SOM, cytochromes, quantitative neighborhoods of atoms, QNA, computational prediction

## Abstract

Metabolism of xenobiotics (Greek *xenos*: exogenous substances) plays an essential role in the prediction of biological activity and testing for the subsequent research and development of new drug candidates. Integration of various methods and techniques using different computational and experimental approaches is one of the keys to a successful metabolism prediction. While multiple structure-based and ligand-based approaches to metabolism prediction exist, the most important problem arises at the first stage of metabolism prediction: detection of the sites of metabolism (SOMs). In this paper, we describe the application of Quantitative Neighborhoods of Atoms (QNA) descriptors for prediction of the SOMs using potential function method, as well as several different machine learning techniques: naïve Bayes, random forest classifier, multilayer perceptron with back propagation and convolutional neural networks, and deep neural networks.

## 1. Introduction

The prediction of the sites of metabolism (SOMs) for the cytochrome P450 family enzymes (in particular, CYP1A2, CYP2C9, CYP2C19, CYP2D6, and CYP3A4 isoforms) plays a pivotal role in drug discovery, as it is possible to generate the chemical structures of metabolites. Knowledge about the structure of metabolites is useful for two reasons: (1) prediction of the toxicity of metabolites; and (2) prediction of the biological activities of potential metabolites for the design of prodrugs.

Currently, there are many experimental and computational approaches to SOM prediction [1,2,3,4,5]. Typically, computational approaches are based on: (1) studies on enzyme-ligand interactions (structure-based methods); and (2) computational modeling of the ligands’ properties based on the modeling sets of known ligands and non-ligands (ligand-based methods).

Structure-based approaches are powerful and widely used methods for estimating intermolecular interactions. When the three-dimensional structure of the target protein is unknown or has low resolution, the application of a structure-based computational approach might be practically impossible [3]. Another limitation of the structure-based design is a variety of ligand-enzyme conformations appearing in the computer-based models, which makes it very difficult to predict several SOMs in one molecule correctly.

Ligand-based approaches have their own limitations, for example, since they are based only on ligand information. Nevertheless, there is a large amount of data on ligands, which are metabolized by cytochrome P450 enzymes, as well as the experimentally-investigated SOMs in a diverse set of the ligands. Accumulated information regarding SOMs in several databases has made it possible to develop models for SOM prediction.

Machine learning approaches, such as artificial neural networks, random forest, naïve Bayes, etc., have become widely used in chemoinformatics in the past few years [6]. The most common application of these machine learning methods is to model the relationship between the chemical structure and the properties which cannot be directly obtained from theoretical chemical methods, for example, using quantum chemistry [6]. From this point of view, machine learning approaches can be applied to drug discovery, in particular for modeling the ADMET (Absorption, Distribution, Metabolism, Excretion, and Toxicity) properties [6].

Recently, several different approaches to ligand-based SOM prediction have been developed, including the application of machine learning algorithms. Recently Tyzack et al. [7] reported an approach to SOM prediction based on probabilistic classifiers such as the naïve Bayesian, and the kernel-based algorithm RASCAL (Random Attribute Subsampling Classification Algorithm) originally developed by the authors. The Parzen-Rosenblatt Window (PRW) was the function created to obtain kernels, which were further used in RASCAL. The full description of the methods is provided in publication [7]. The developed algorithms were applied to predict the SOMs for the modeling sets of chemical compounds metabolized by CYP2C9, CYP2D6, and CYP3A4. The authors compared their approach to those developed previously, such as Xenosite (for details, see [8]), RS-predictor [9], and SMARTCyp [10]. Comparison with the other approaches was performed using the Top-2 metrics: the percentage of structures in the modeling set where a SOM was identified in the top two predictions. The accuracy of prediction was comparable to the reference methods.

Rudik et al. [11] developed an application of the naïve Bayes based approach, implemented in the PASS (Prediction of Activity Spectra for Substances) program for SOM prediction. Here, the authors proposed modifications of previously developed multilevel neighborhoods of atoms (MNA) descriptors [12], where each atom-SOM was specifically labeled in contrast to non-SOM atoms during the generation of the descriptors. The comparison of the approach with SMARTCyp and RS-web-predictor were provided based on the area under the received operating characteristic curve (AUC) and Top-1, Top-2, and Top-3 metrics. The comparison of the accuracies concluded that the PASS-based approach provided a similar or higher accuracy than the reference methods in most cases.

We designed a simple approach for the SOM/non-SOM classification, which was based on the application of QNA descriptors [13] and various machine-learning algorithms. QNA descriptors are pairs of *P* and *Q* values calculated for each atom of a molecule based on its connectivity matrix and the standard values of the ionization potential (IP) and electron affinity (EA) of the atoms. They describe each of the atoms in a molecule, and, at the same time, each of the *P* and *Q* values depend on the whole composition and structure of a molecule. The previously developed ligand-based approaches [7,11] were mainly based on the use of either fragmental descriptors (substructures) or fingerprints, generated for each atom of the chemical structure and specifically tagged if a certain atom corresponded to the SOM. In most cases, the SOM corresponds to a single atom, so the atom-centered QNA descriptors seem best for representation of SOMs. Labels of SOM and non-SOM for each atom of a molecule can be generated automatically based on the SDF file used as input. We proposed to use the data on SOM and non-SOM heavy atoms only.

## 2. Results

We used the modeling sets compiled according to (i) the specific type of metabolizing reaction; and (ii) both the name of the isoform and reaction type that it catalyzes. The preparation of modeling sets is described in detail in the Material and Methods section. In total, seven sets of the compilation type were created including: (i) aliphatic-hydroxylation, aromatic hydroxylation, *C*-oxidation, *N*-dealkylation, *N*-oxidation, *O*-dealkylation, and *S*-oxidation. For the following description of the results obtained using compilation type (ii), we selected five of the most representative datasets for more compact representation, and prepared the following modeling sets: CYP1A2-*C*-oxidation, CYP1A2-aliphatic hydroxylation, CYP2C9-*S*-oxidation, CYP3A4-*C*-oxidation, and CYP2C19-*O*-dealkylation.

The following machine learning algorithms were investigated: naïve Bayes (NB) and random forest classifiers (RF), as well as several different artificial neural network classification approaches (radial basis function (RBF) network, multilayer perceptron (MLP), and convolutional neural network (Conv. NN)).

**Figure 1:**
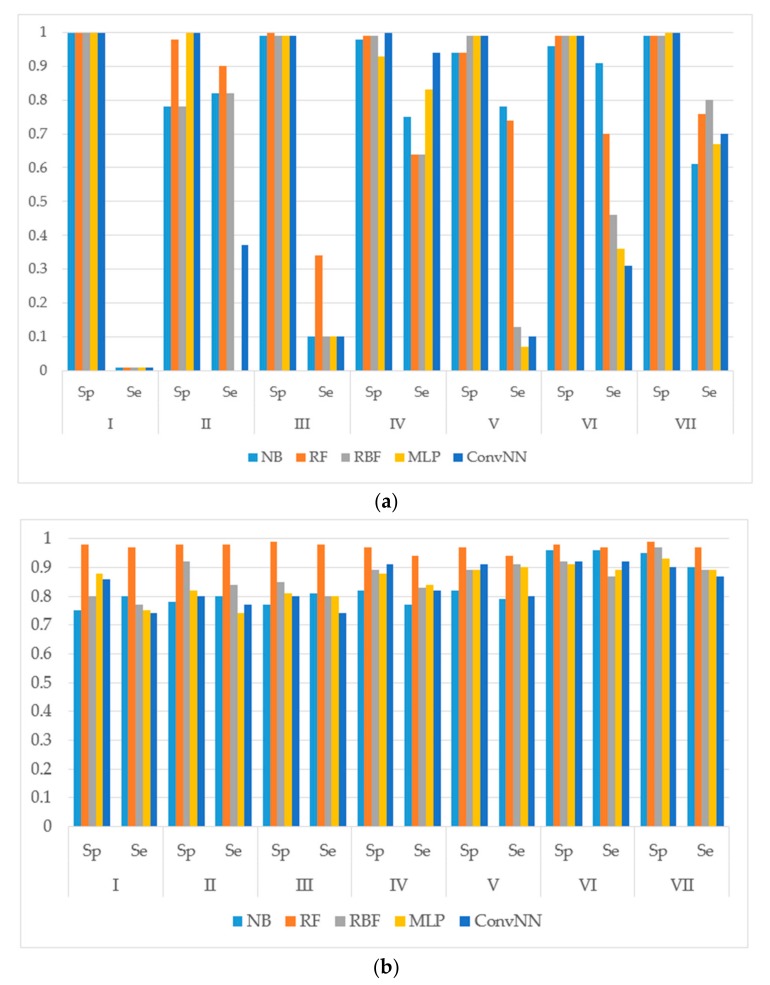
Sensitivity (Sp) and specificity (Se) of the classification models for SOM predictions of the datasets compiled according to the type of metabolizing reaction: (**a**) datasets before balancing; and (**b**) balanced datasets. X-axis: Roman numerals correspond to the following reaction types I: aliphatic-hydroxylation; II: aromatic hydroxylation; III: *C*-oxidation; IV: *N*-dealkylation; V: *N*-oxidation; VI: *O*-dealkylation; and VII: *S*-oxidation. Machine learning algorithms: RF: random forest; RBF: radial basis function network; MLP: multilayer perceptron; Conv. NN: convolution neural network; AUC: area under the received operating characteristic curve. Y-axis: Sp is obtained as: TP/(TP + FN). Se is obtained as TN/(TN + FP). TP: Number of true positive predicted samples; FP: number of false positive; TN: number of true negative; FN: number of false negative.

**Figure 2:**
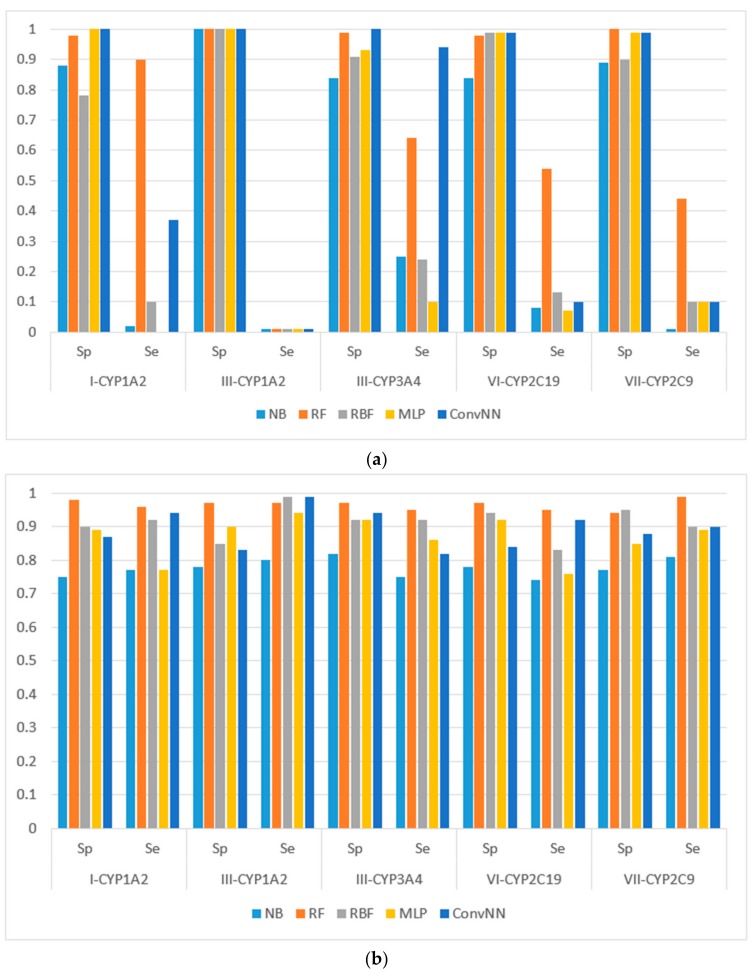
Sensitivity (Sp) and specificity (Se) of the classification models for SOM predictions of the datasets compiled according to the type of both metabolizing reaction and enzymes: (**a**) datasets before balancing; and (**b**) balanced datasets. X-axis: Roman numerals correspond to the following reaction types: I-CYP1A2: CYP1A2-aliphatic hydroxylation; III-CYP1A2: CYP1A2-*C*-oxidation; III-CYP3A4: CYP3A4-*C*-oxidation; VI-CYP2C19: CYP2C19-*O*-dealkylation; VII-CYP2C9: CYP2C9-*S*-oxidation. Machine learning algorithms: RF: random forest; RBF: radial basis function network; MLP: multilayer perceptron; Conv. NN: convolution neural network; AUC: area under the received operating characteristic curve. Y-axis: Sp = TN/(TN + FP), Se = TP/(TP + FN), TP is the number of true positives, FP is the number of false positives, TN is the number of true negatives, and FN is the number of false negatives.

We calculated the performances of the models using different machine learning methods implemented in Weka 3.6.12 [14]. The performances of models were estimated using the following metrics: specificity (Sp), sensitivity (Se), balanced accuracy (BA) and area under the received operating characteristic curve (AUC). The results for the modeling sets compiled based on the name of the reaction are presented in Figure 1. The results for the modeling sets compiled according to the name of enzyme and the type of metabolizing reaction are presented in Figure 2.

The average performances (AUC and BA) for each machine learning classifier and standard deviations for the set of values obtained for each modeling set using one machine learning method are given in Table 1.

## 3. Discussion

It is noteworthy to mention that AUC values are, in general, higher for all the modeling algorithms comparing to those of balanced accuracy (BA, BA = (sensitivity + specificity)/2) values. In general, BA values are more sensitive than AUC, therefore, we prefer to use BA values to compare and discuss the modeling results.

We made the following observations based on the values of sensitivity, specificity, and BA. First, the performances of the models for the modeling sets compiled according to the reaction type were significantly higher in comparison to the compilation based on both the name of the enzyme and the reaction type. The small size of the modeling sets in the second compilation type can be a cause of the relatively low performance of the models.

Second, for the initial imbalanced modeling sets, random forest classifier gave the best performances; therefore, these methods were more stable and predictive in comparison to the methods based on artificial neural networks when imbalanced datasets are modeled. Therefore, these approaches might be the methods of choice for modeling imbalanced modeling sets with a low number of independent variables (for instance, in our case, only two QNA descriptors: *P* and *Q*). In most cases, the radial basis function classifier had the best performance among the several artificial neural networks models implemented in Weka. This fact may be explained by the pre-training clusterization (i.e., unsupervised classification) before input into the network. In general, the second type of modeling set compilation (i.e., by reaction type and by isoform) leads to a significant increase of accuracy, despite the lower sizes of datasets.

Third, we concluded that the random forest classifier had the best performance for both the balanced and imbalanced modeling sets, despite using only two descriptors (floating point values) as the independent variables of the model. The methods based on artificial neural network, in general, yielded better performances when compared to the random forest classifier only for the balanced modeling sets.

### Comparison with Other Approaches

We applied the machine learning methods based on the use of QNA descriptors to the datasets containing CYP2C9, CYP2D6, and CYP3A4, which were provided by Tyzack et al. [7], because (1) the datasets used in that study were available; and (2) the approach of Tyzack et al. was the most similar ligand-based approach with the application of machine-learning methods.

We balanced these datasets as described in the Section 4. In [7], the leave-one-out cross validation procedure was used. We decided to obtain the AUC values by using the leave-one-out cross validation to compare the results with those obtained by Tyzack et al. We also decided not to use the Top-2 metrics in our approach as it does not allow the correct estimation of false positive results. Both the average and the best values of the performances for each isoform (balanced datasets) are given in Table 2.

In our QNA-based method, the best performances were obtained for random forest (all isoforms). The best performances in [7] were obtained from PRW (CYP2C9), PRW, and RASCAL (CYP2D6), as well as PRW (CYP3A4).

The given results allowed to conclude that the QNA-based approach obtained comparable results when compared to the most similar approach based on the machine-learning methods, reported previously in [7].

Furthermore, we compared the average performances of the method presented in this work (for balanced datasets) with the results that have been reported in previous studies [7,8,9,10,11]. The average performances are provided in Table 3. We also compared our results to an earlier published study of Rudik et al. (Table 3).

Overall, the performance comparison showed that, in general, our method outperformed the prediction accuracy of most of the methods cited when the training datasets were balanced before prediction. The method of choice for SOM prediction based on QNA descriptors is the random forest classifier. Therefore, the advantages of the presented method included the possibility of obtaining good performances, as well as the ease of generating QNA descriptors for both SOM and non-SOM atoms (easiness of SOM and non-SOM labeling). The low power of the computational units needed to build the models that occur due to a relatively small dimensionality can also be considered as an advantage of our method. However, the relatively low performances for the models built based on the initial dataset before being balanced is definitely a disadvantage of the presented method.

## 4. Materials and Methods

We used the modeling sets compiled from several databases including DrugBank (HMDB), ChEMBLdb, ADME DB (Fujitsu), and Metabolite (Biovia); they contained the structural formulae of the chemical compound and the experimental data of sites of metabolism in it. The collected modeling sets contained the structural formulae of the ligands of the main cytochrome P450 isoforms that metabolize small molecules: CYP1A2, CYP2C9, CYP2C19, CYP2D6, and CYP3A4 (the number of the substrates are given in Table 4; data sets of chemical compounds are given in Supplementary Materials).

We studied how compiling the modeling sets according to a specific reaction type may—influence the prediction performances. The reaction types considered were: aliphatic hydroxylation, aromatic hydroxylation, *N*-, *S*-, *C*-oxidation, *N*- and *O*-dealkylation. Classification by metabolizing reactions was based on the classifications used in the databases where the datasets were obtained. The set of aliphatic hydroxylation reactions and the set of *C*-oxidation were distinguished. The term “aliphatic hydroxylation” describes the reactions forming hydroxyl groups on carbon atoms, which are not in aromatic cycles; the term “*C*-oxidation” describes reactions forming carboxyl or carbonyl groups on carbon atoms.

We have created several subsets according to (i) the general type of metabolizing reaction; and (ii) the type of reaction catalyzed by the specific isoform. Therefore, the prediction was made for the modeling sets corresponding to (i) the type of reaction catalyzed by the isoform, and (ii) a particular isoform of the cytochrome and the reaction that it catalyzes. The number of substrates with SOMs in each subset created as described above is given in Table 4.

**Figure 3:**
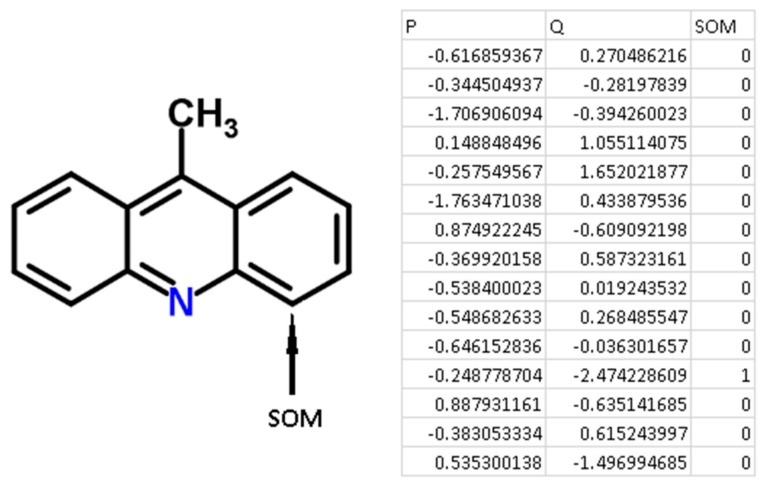
An example of the generation of QNA descriptors for the molecule 9-methylacridine.

For each atom of the structures from the chemical modeling sets, normalized *P* and *Q* values (QNA descriptors) were calculated. Each SOM-atom had the value “1”, non-SOM atoms had the value of “0”. These values of SOM were used as the target variable in the models, while *P* and *Q* values were independent variables. An example of the descriptors generated for 9-methylacridine, with antibacterial and antitumor activity is given in Figure 3.

There are some advantages of using QNA descriptors for the purposes of SOM prediction using machine learning approaches. First, the advantage derived from the principles of generating QNA descriptors. Each atom (SOM and non-SOM) corresponds to one of the QNA descriptors, the values of *P* and *Q*. Therefore, it is easy to generate descriptors fully associated with each atom of a molecule and label SOM and non-SOM atoms. Any structural descriptors (i.e., Extended Connectivity Fingerprints, ECFP, molecular signatures, MNA descriptors) typically represent a part of a molecule and include the set of atoms in each descriptor. Specific technical efforts must be undertaken to label SOMs and non-SOMs. Second, there is no need to select the most important features (descriptors) for modeling due to the small dimensionality of the QNA descriptors used in the study. Third, the size of the training, test, and validation sets do not need to be extremely large because of the relatively low number of features used in the model.

There is a significant imbalance in the modeling sets due to the nature of QNA generation for SOM prediction: the ratio of the number of SOMs to the number of non-SOMs does not exceed 0.05 in all modeling sets. Therefore, the modeling sets that were created as described above were significantly imbalanced. This is the result of representation of each atom as a single point in the modeling set: the number of SOM-atoms was significantly lower than the number of non-SOM atoms as typically, only one or two sites of metabolism occur in the molecule. Therefore, we had to balance the modeling sets to obtain the more predictive models. To balance the modeling sets, we used an approach to synthetically multiply the minor class instances (SOM atoms), realized in Python (Synthetic Minority Oversampling Technique, SMOTE). In that algorithm, the finding k-nearest neighbors for observations of minor class and generating similar samples in the feature space lead to oversampling of the minor class [15]. We used the following parameters of SMOTE algorithm. The number of neighbors for each minority class sample was five. After oversampling the ratio of minority class samples (SOM) to samples of majority class (non-SOM) is one. Typically, the number of SOM after resampling was approximately 48–50% of the total training set. The level of resampling (ration of SOM to non-SOMs samples) was consistent across all datasets. To avoid overfitting, we used oversampling only for the training sets, but did not apply oversampling for validation and test sets.

We decided to study the well-known machine learning methods applied to QNA-based SOM prediction: naïve Bayes and random forest classifiers, as well as several different artificial neural network classification approaches (radial basis function (RBF) network, multilayer perceptron (MLP), and convolutional neural network (Conv. NN)). We used Weka 3.6.12 [14] to build the models. Accuracy of prediction was calculated using the standard for the classification model metrics: AUC, sensitivity, specificity, and BA. All modeling sets were divided into training and test sets in a proportion of 2:1. This procedure was repeated five times. After five-fold training of the models and obtaining results for the test sets, the results were averaged.

During the training, we empirically determined the parameters of each Weka classifier that correspond to the highest performances of models: random forest, RBF network, MLP, and Conv. NN. The parameters were initially determined for both imbalanced and balanced sets. Afterwards, we have determined the best parameters for balanced datasets since the performances of the initial imbalanced modeling sets were comparatively low for all considered reaction and isoform types. In most cases, the parameters corresponded to the best accuracies of balanced datasets were the same as the best accuracies for imbalanced datasets. For further use, a parameter for the random forest classifier was applied: 100 iterations and 70% of the modelling set that underwent bootstrap aggregating (bagging). During evaluation of different parameters (100, 500, 1000 and 2000 iterations and 80%, 90% and 100% of dataset that undergo bagging), we concluded that an increase in the number of trees did not lead to a significant increase in accuracy for both balanced and imbalanced datasets. The other parameters of random forest classifier were used in Weka by default. The parameters recommended for the use of the RBF network were obtained from study [16], and we used two clusters of the data as the input data for the network; the activation threshold of each net unit = 10^−8^. Parameters of the MLP were selected as two hidden layers, 10 neurons in each one; and hyperbolic tangent function was used as an activation function; other parameters were the Weka default parameters. We have estimated the accuracy obtained by changing the number of hidden layers from one to three. It is known that, in principle, one hidden layer can be useful for nonlinear approximation of any nonlinear function. In our experiment, the accuracy of prediction was not significantly changed when the number of hidden layers had been increased from one to three for initial imbalanced modeling sets. However, for balanced modeling sets, the highest accuracy was achieved in case of hidden layers’ value of two. For the convolution neural network, we used the parameters which were the best ones for obtaining the highest accuracies: two hidden layers with 100 convolution neurons in each. Each convolution neuron contained two hidden layers with five neurons in each layer, with rectifier linear unit function as an activation function. We would like to point out that increasing either the number of the hidden layers or the number of neurons in each layer did not significantly increase the performances.

Since the datasets from the previously developed approach described in publications [7,11] are available, we also decided to apply the machine learning methods based on the use of QNA descriptors to these datasets to perform comparison of our method with the other published ones.

## 5. Conclusions

A new approach based on quantitative neighborhoods of atoms (QNA) descriptors using machine learning methods was presented. We compared five different machine learning methods including the following: naïve Bayes, random forest, radial basis function network, multilayer perceptron, and convolutional neural network (implemented in Weka). Considering that the number of SOMs was significantly lower than the number of non-SOMs, the modeling sets containing QNA descriptors used in our study were highly imbalanced. We applied the models to both the initial sets and the balanced datasets. The comparison of the machine-learning methods allowed us to conclude that the random forest classifier gave the best performances for both the balanced and imbalanced datasets. Comparison of the approach described in this paper with a previously-developed one based on machine learning methods allowed us to conclude that the QNA-based approach using machine learning yielded performances comparable to those reported earlier by Tyzack et al. [7]. Therefore, the use of QNA descriptors in machine learning methods may be applicable to the purposes of metabolism site prediction.

## Figures and Tables

**Table 1:** Average values of performances obtained for the (**a**) initial datasets before balancing and (**b**) balanced modeling sets using multiple machine learning classifiers.

(**a**)				
**Parameter**	**i ^1^**		**ii ^2^**	
	**AUC (SD)**	**BA (SD)**	**AUC (SD)**	**BA (SD)**
Naïve Bayes	0.90 (0.15)	0.76 (0.17)	0.85 (0.02)	0.48 (0.04)
RF	0.90 (0.05)	0.75 (0.16)	0.92 (0.02)	0.78 (0.19)
RBF	0.87 (0.09)	0.69 (0.15)	0.87 (0.04)	0.51 (0.05)
MLP	0.86 (0.12)	0.63 (0.16)	0.87 (0.09)	0.51 (0.18)
Conv. NN	0.87 (0.15)	0.67 (0.17)	0.86 (0.08)	0.65 (0.09)
(**b**)				
**Parameter**	**i ^1^**		**ii ^2^**	
	**AUC (SD)**	**BA (SD)**	**AUC (SD)**	**BA (SD)**
Naïve Bayes	0.96 (0.007)	0.83 (0.07)	0.92 (0.008)	0.78 (0.02)
RF	0.99 (0.005)	0.97 (0.01)	0.99 (0.005)	0.97 (0.008)
RBF	0.95 (0.007)	0.88 (0.06)	0.97 (0.04)	0.91 (0.016)
MLP	0.97 (0.009)	0.85 (0.03)	0.92 (0.19)	0.87 (0.016)
Conv. NN	0.965 (0.007)	0.84 (0.05)	0.94 (0.08)	0.92 (0.13)

^1^ The performances obtained for the datasets compiled according to the type of metabolizing reaction; ^2^ The performances obtained for the datasets compiled according to both the type of metabolizing reaction and enzymes. RF: random forest; RBF: radial basis function network; MLP: multilayer perceptron; Conv. NN: convolution neural network; AUC: area under the received operating characteristic curve; BA: balanced accuracy.

**Table 2:** Comparison of the performances of QNA-based approach and the results reported in Tyzack et al. showing the average and the best AUC values obtained based on the leave-one-out cross-validation.

Isoform of Cytochrome	AUC_av_^1^	SD_AUC_^2^	AUC_max_^3^	AUC_[ref]_^4^
CYP2C9	0.91	0.07	0.99	0.97
CYP2D6	0.90	0.09	0.99	0.97
CYP3A4	0.91	0.07	0.97	0.96

^1^ Average AUC values obtained in the methods, implemented in Weka (five-fold random division into training, test sets, and validation sets (for MLP, ConvNN only)); ^2^ Standard deviation for the AUC values; ^3^ The highest AUC value; ^4^ AUC values for methods implemented in Tyzack et al. that have been previously published.

**Table 3:** Comparison of the performances of the QNA-based approach and the results reported in Rudik et al., 2014. The average and best AUC values in our method were obtained by means of random division into the training and test sets in a proportion of 2:1.

Isoform of Cytochrome	AUC_av_^1^	SD_AUC_^2^	AUC_max_^3^	AUC_[ref]_^4^
CYP1A2	0.86	0.12	0.98	0.83
CYP2C9	0.85	0.08	0.94	0.87
CYP2C19	0.82	0.09	0.92	0.76
CYP2D6	0.87	0.09	0.94	0.83
CYP3A4	0.85	0.09	0.95	0.85

^1^ Average AUC values obtained in the methods, implemented in Weka; ^2^ Standard deviation for the AUC values; ^3^ The highest AUC value; ^4^ AUC values for methods implemented In Rudik et al. that were previously published.

**Table 4:** The number of chemical structures in the modeling sets used in the study.

Isoform of Cytochrome	I	II	III	IV	V	VI	VII	N	N1
CYP1A2	201	31	179	55	108	44	165	463	803
CYP2C9	165	167	25	125	19	93	35	268	643
CYP2C19	172	122	13	138	23	87	49	369	607
CYP2D6	167	200	13	215	34	149	36	466	846
CYP3A4	474	269	179	419	95	207	112	570	1004

The designations of columns are: I: aliphatic hydroxylation; II: aromatic hydroxylation; III: *C*-oxidation; IV: *N*-dealkylation; V: *N*-oxidation; VI: *O*-dealkylation; VII: *S*-oxidation; N: number of unique substrates; and N1: number of records with the labeled SOM.

## Supplementary Materials

Supplementary Materials are available online. Datasets with chemical structures and datasets with QNA descriptors and SOM labels are given in the Supplementary Materials.
